# Long non-coding RNA MIAT serves as a biomarker of fragility fracture and promotes fracture healing

**DOI:** 10.1186/s13018-024-04824-7

**Published:** 2024-06-08

**Authors:** Chao Yu, Binbin Chen, Hui Su, Yiqun Yang

**Affiliations:** 1 Department of Orthopedics, Liaocheng People’s Hospital, No. 67, West Dongchang Road, Liaocheng, 252000 China; 2 Department of Nephrology, Liaocheng People’s Hospital, Liaocheng, 252000 China; 3 Department of Oncology, Liaocheng People’s Hospital, Liaocheng, 252000 China

**Keywords:** MIAT/miR-181a-5p, Osteoporosis, Fracture healing, MC3T3-E1

## Abstract

**Background:**

Fragility fracture is common in the elderly. Osteoblast differentiation is essential for bone healing and regeneration. Expression pattern of long non-coding RNA MIAT during fracture healing was examined, and its role in osteoblast differentiation was investigated.

**Methods:**

90 women with simple osteoporosis and 90 women with fragility fractures were included. Another 90 age-matched women were set as the control group. mRNA levels were tested using RT-qPCR. Cell viability was detected via CCK-8, and osteoblastic biomarkers, including ALP, OCN, Collagen I, and RUNX2 were tested via ELISA. The downstream miRNAs and genes targeted by MIAT were predicted by bioinformatics analysis, whose functions and pathways were annotated via GO and KEGG analysis.

**Results:**

Serum MIAT was upregulated in osteoporosis women with high accuracy of diagnostic efficacy. Serum MIAT was even elevated in the fragility fracture group, but decreased in a time manner after operation. MIAT knockdown promoted osteogenic proliferation and differentiation of MC3T3-E1, but the influences were reversed by miR-181a-5p inhibitor. A total of 137 overlapping target genes of miR-181a-5p were predicted based on the miRDB, TargetScan and microT datasets, which were mainly enriched for terms related to signaling pathways regulating pluripotency of stem cells, cellular senescence, and osteoclast differentiation.

**Conclusions:**

LncRNA MIAT serves as a promising biomarker for osteoporosis, and promotes osteogenic differentiation via targeting miR-181a-5p.

## Background

Fragility fracture refers to a kind of fracture that occurs after a non-traumatic or minimal wound [1]. Minor trauma is usually caused by a fall on the level or at the height of the body’s center of gravity. Osteoporosis causes weak bones and increases the risk of fractures [2]. Fragility fractures are more common in the elderly, and due to the unique physiological characteristics of women, the probability of fragility fractures is higher [3, 4]. Fragility fracture often occurs in the hip and spine, followed by wrists and shoulders [5]. Elderly hip fragility fractures have a high mortality and disability rate, and the resulting injuries are often fatal [6]. In addition, fragility fracture imposes tremendous financial burden on society. Recently, the risk prediction of fracture in osteoporosis heavily depends on dual-energy X-ray absorptiometry (DXA) measurement at the lumbar spine and/or femoral hip [7]. However, it showed a limited power in differentiating individuals at high risk of fragility fractures.

Long non-coding RNA (lncRNA) is the most abundant type of ncRNA in living organisms. Accumulating lncRNAs have been found to regulate cell function, affect cell fate, organ development and the occurrence and development of diseases [8]. There are lncRNAs in the organism that can regulate the differentiation of osteoblasts, which are considered to be potential targets to intervene osteoporosis, as well as fragility fracture [9]. Myocardial infarction-associated transcript (MIAT) is a highly conserved lncRNA. Recently, MIAT has been identified to participate in multiple diseases, such as microvascular dysfunction, myocardial infarction and cancer [10, 11]. Notably, high expression of lncRNA MIAT has been detected in the peripheral blood mononuclear cells of post-menopausal osteoporosis patients, with the underlying mechanism of the inflammatory response [12]. Moreover, the function of lncRNA MIAT in human adipose-derived stem cells (hASCs) has been investigated, and MIAT knockdown is determined to enhance osteogenic differentiation of hASCs both in vitro and in vivo [13]. However, the precise role of lncRNA MIAT in fragility fracture has not been elucidated.

Therefore, the expression pattern of lncRNA MIAT was tested in patients with osteoporosis and fragility fracture, and its diagnostic value was explained. Moreover, the precise role of lncRNA MIAT in osteoblast proliferation and differentiation was also explored in MC3T3-E1 cells. Furthermore, the downstream lncRNA-miRNA-mRNA network was preliminarily studied. It is believed that a better understanding of lncRNA regulatory networks will help shed more light on the nature of fragility fracture and ultimately facilitate improvements in lncRNA-directed diagnosis and treatment.

## Methods

### Human participant recruitment

Patients enrolled in the study were females from Liaocheng People’s Hospital who were diagnosed with osteoporosis. Finally, a total of 180 cases with recruited, including 90 women with simple osteoporosis and 90 patients with fragility fracture. The diagnosis of osteoporosis was based on the WHO criteria of bone mineral density (BMD) T-sore ≤ -2.5 [14]. Patients with the following conditions were excluded from the study: (1) with pathological fracture; (2) old fracture (more than 14 days); (3) had a history of autoimmune diseases or bone metastasis; (4) taking anti-osteoporotic agents. Another 90 age-matched women were set as the control group. The nature and possible consequences were informed to all participants. After that, 5 mL whole blood samples were taken from each participant post-operative, which were immediately applied for serum isolation. For fragility fracture cases, 5 mL blood samples were also collected at 24 h post-operation. In addition, blood was taken every other week for four weeks. Informed consent was obtained from all individual participants included in the study. Approval was obtained from the ethics committee of Liaocheng People’s Hospital. The procedures used in this study adhere to the tenets of the Declaration of Helsinki.

### Cell culture and treatment

MC3T3-E1 cells (cat no. CRL-2593) were acquired from American Type Culture Collection (Rockville, MD, USA), which were cultured in DMEM containing 10% fetal bovine serum maintained in a 37℃, 5% CO2 incubator. Then cell differentiation was induced using 300 ng/mL bone morphogenetic protein 2 (BMP2) for two weeks. At 0, 7 and 14 days after induction, the cell differentiation was detected through testing osteoblastic biomarkers, including ALP, OCN, Collagen I, and RUNX2.

### ELISA assay

The concentration of osteoblastic biomarkers, including ALP (cat no. E-EL-H6221), OCN (cat no. E-AB-16,278), Collagen I (cat no. E-AB-10,155), and RUNX2 (cat no. E-AB-30,103) in MC3T3-E1 cell supernatants were quantified by a commercially available ELISA Kit from Elabscience. Briefly, protein standards were diluted and added to the plate, then HRP-coupled detection antibodies were added. The absorbance at 450 nm was measured after adding the termination solution.

### Cell transfection

MC3T3-E1 cells were inoculated into 6-well plates and cultured overnight. Small interfering (si) of MIAT (cat no. siG151109052037-1-5) and its negative control (si-NC; cat no. siN0000002-1-5), miR-181a-5p mimic (cat no. miR10000256-1-5) or inhibitor (cat no. miR20000256-1-5), and mimic NC (cat no. miR1N0000001-1-5) or inhibitor NC (cat no. miR2N0000001-1-5) were received from Ribobio. Then 100 nM si-MIAT or si-NC, or 50 nM miR-181a-5p mimic or inhibitor, or 50 nM mimic NC and inhibitor NC were mixed with Lipofectamine 2000 respectively and added to 6-well plates. Cells that did not receive transfection were set as the control group.

### Reverse transcription-quantitative PCR (RT-qPCR)

The miRNeasy Serum/Plasma Kit (cat no. 217184) and miRNeasy Kit (cat no. 217084; Qiagen) were applied for the extraction of total RNA from patients’ serum or cell lines, respectively. And RNA purity and concentration were checked using a NanoDrop (Thermo, USA), and the absorbance ratio of 260/280 nm in 1.8-2.0 indicated good-quality RNA. Then complementary DNA was transcribed from 5 µg RNA using the First Strand cDNA Synthesis Kit (Invitrogen; cat no. 12328032) and miScript II RT kit (Qiagen; cat no. 218161). Then RT-qPCR amplification was done using SYBR Green qPCR SuperMix (Invitrogen; cat no. 11744100) for lncRNA MIAT or miScript SYBR Green PCR Kit (Qiagen; cat no. 218073) for miR-181a-5p and mixed with primers, cDNA, and ddH_2_O. The following thermocycling conditions were used for the PCR: initial denaturation at 94˚C for 2 min, followed by 40 cycles of 94˚C for 20 sec, 60˚C for 34 sec. After amplification, a melting curve was generated to evaluate the specificity of PCR products at the end of each PCR cycle. And the amplification of only one product in qRT-PCR was confirmed by a melting curve analysis. Relative levels of lncRNA and miRNA were calculated using GAPDH and U6 as internal controls and normalized and quantified by the 2^−ΔΔCt^ method. The primers used were as follows: MIAT forward 5’- CACAAAGAGCCCTCTGCACTAG − 3’ and reverse 5’- TGGCCACATGAACGTGTCTG − 3’ (94 bp, GenBank accession no. NG_016621), miR-181a-5p forward 5′-CCGCGAACATTCAACGCTGTCG3′ and reverse 5′-ATCCAGTGCAGGGTCCGAGG-3′ (72 bp, GenBank accession no. NC_000009), GAPDH forward 5′-TGTTCGTCATGGGTGTGAAC-3′ and reverse 5′-ATGGCATGGACTGTGGTCAT-3′ (154 bp, GenBank accession no. AF261085), and U6 forward 5′-CTCGCTTCGGCAGCACA-3′ and reverse 5′-AACGCTTCACGAATTTGCGT-3′ (96 bp, GenBank accession no. NR_004394).

### CCK-8 assay

2 × 10^3^ MC3T3-E1 cells were resuspended to gain a 100 µl resuspension, which was added to a 96-well plate. After incubation for three days, the cell viability was detected. In brief, the cell medium was replaced by mixing Cell Counting Kit 8 (CCK-8; cat no. 40203ES76, Biotechnology) with DMEM in a 1:9 ratio every 24 h for three consecutive days. The absorbance value at OD 450 nm was tested after continuing incubation at 37℃ for 1 h.

### Luciferase reporter gene assay

As the ENCORI online database predicted, lncRNA MIAT could bind to miR-181a-5p. The validation of miR-181a-5p bindings to the 3’UTR of MIAT was conducted via dual luciferase reporter (DLR) assay. Firstly, MIAT fragments containing miR-181a-5p binding sequences or fragments with site-specific mutations were first amplified and subcloned into the luciferase reporter vector pmirGLO to construct MIAT wild type (WT-MIAT) or MIAT mutant type (MUT-MIAT) recombinant luciferase plasmids (cat no. CL414-01; Biomed). MC3T3-E1 cells were inoculated into a 6-well plate and the recombinant plasmid was transfected with a miR-181a-5p mimic or inhibitor. The luciferase activity was detected after lysis of cells.

### Go and KEGG analysis

Downstream target genes of miR-181a-5p were predicted using miRDB (Target Score > 80), TargetScan (Total context + + score < -0.30), and microT (interaction_score > 0.9). Then Venn diagrams were conducted for the visualization of overlapping genes in the three databases. The enriched terms and pathways of the overlapping genes were annotated using Gene Ontology (GO) and Kyoto Encyclopedia of Genes and Genomes (KEGG) pathway analyses, respectively.

### Statistical analysis

Statistical data were presented as mean ± standard deviation (SD) or frequency (n), which were analyzed with GraphPad Prism 9.0 and SPSS 23.0 software. Each experiment had three technical repetitions. One-way analysis of variance (ANOVA) was used to compare differences among multiple groups more than 2, followed by Tukey’s test for post-hoc comparisons, while the students’ t test was for difference comparison between two groups. *P* < 0.05 was considered a statistically significant difference. Receiver operating characteristic curve (ROC) was plotted to evaluate the diagnostic efficacy of MIAT in osteoporosis at a significant value less than 0.05.

## Results

### Characteristics of the study subjects

The demographic and clinical profiles of the three study groups are depicted in Table 1. There was no statistically significant difference in age and BMI (*P* > 0.05). Values of Vitamin B12, 25-(OH) Vitamin D and T scores differed significantly among the three groups, and high values were detected in cases in osteoporosis and fragility fracture groups compared to the control group, in which fragility fracture had the lowest values for each item. For the fragility fracture group, the types were recorded, mainly including hip, forearm/wrist, and vertebral, in which hip fracture had the highest proportion.

**Table 1:** The demographic and clinical data of the study population

Items	Control group (*n* = 90)	Osteoporosis (*n* = 90)	Fragility facture (*n* = 90)	*P* value
Age	65.57 ± 5.32	65.20 ± 4.97	65.46 ± 4.81	0.882
BMI, kg/m^2^	23.59 ± 2.82	24.35 ± 3.05	24.49 ± 2.95	0.090
Vitamin B12, pg/mL	709.29 ± 129.96	495.11 ± 96.50	469.63 ± 90.48	< 0.001
25-(OH) Vitamin D, ng/mL	40.04 ± 5.09	25.35 ± 3.00	15.87 ± 3.03	< 0.001
T score	-0.75 ± 0.15	-2.87 ± 1.06	-3.18 ± 1.27	< 0.001
Types of fracture, n				
Hip	-	-	51	
Forearm/wrist	-	-	28	
Vertebral	-	-	3	
Others	-	-	8	

### Expression of lncRNA MIAT in the study groups

**Fig. 1:**
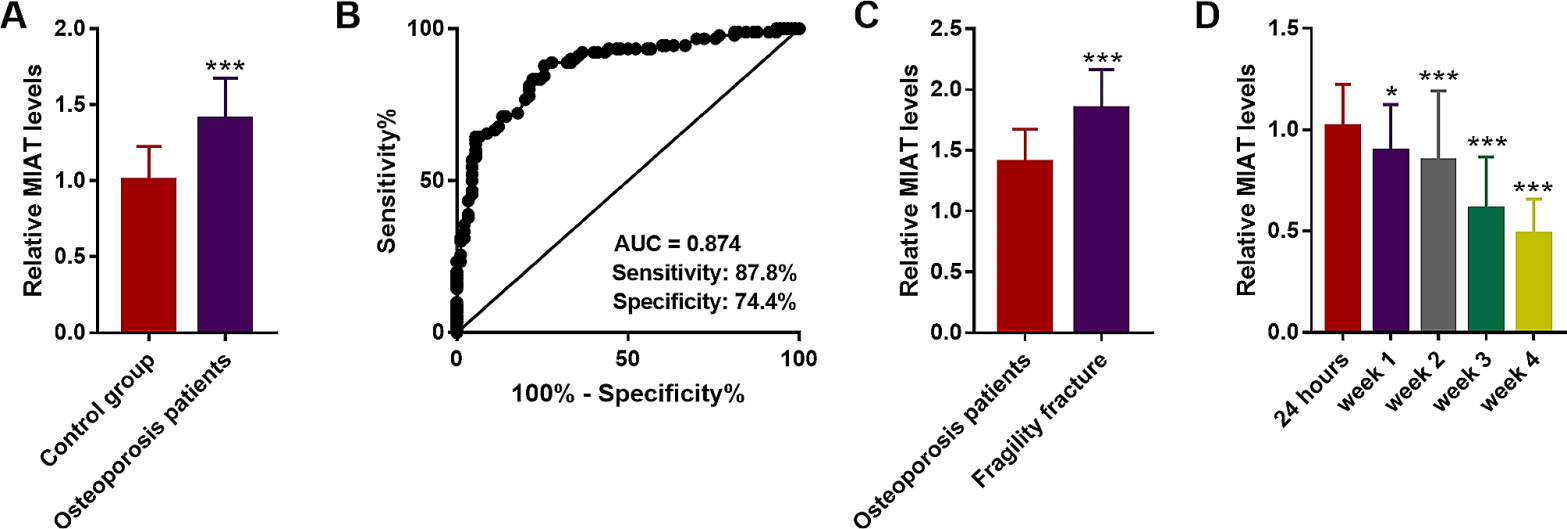
Expression of lncRNA MIAT in osteoporosis and fragility fracture women. **A**. A distinctly upregulated trend was detected in osteoporosis women compared with the control group. **B**. ROC curve of MIAT for osteoporosis. **C**. Serum MIAT levels were even elevated in the fragility fracture group. **D**. Serum MIAT decreased in a timely manner after the operation. * *P* < 0.05, *** *P* < 0.001

Figure 1A presented the quantification of serum MIAT in osteoporosis women by RT-qPCR, and a distinctly upregulated trend was detected compared with the control group (*P* < 0.05). ROC curve performed that lncRNA MIAT had high accuracy of diagnostic efficacy, in which the AUC was 0.874 for osteoporosis, with the sensitivity of 87.8% and the specificity of 74.4% (Fig. 1B). The outcome illustrated the potential of lncRNA MIAT as a valuable tool for early osteoporosis diagnosis. In comparison with the osteoporosis group, serum MIAT levels were even elevated in the fragility fracture group (*P* < 0.05, Fig. 1C). After the operation, serum MIAT decreased in a timely manner, and reached the lowest level at 4 weeks (Fig. 1D), demonstrating that MIAT may participate in the fracture healing.

### MIAT knockdown promotes osteogenic differentiation

**Fig. 2:**
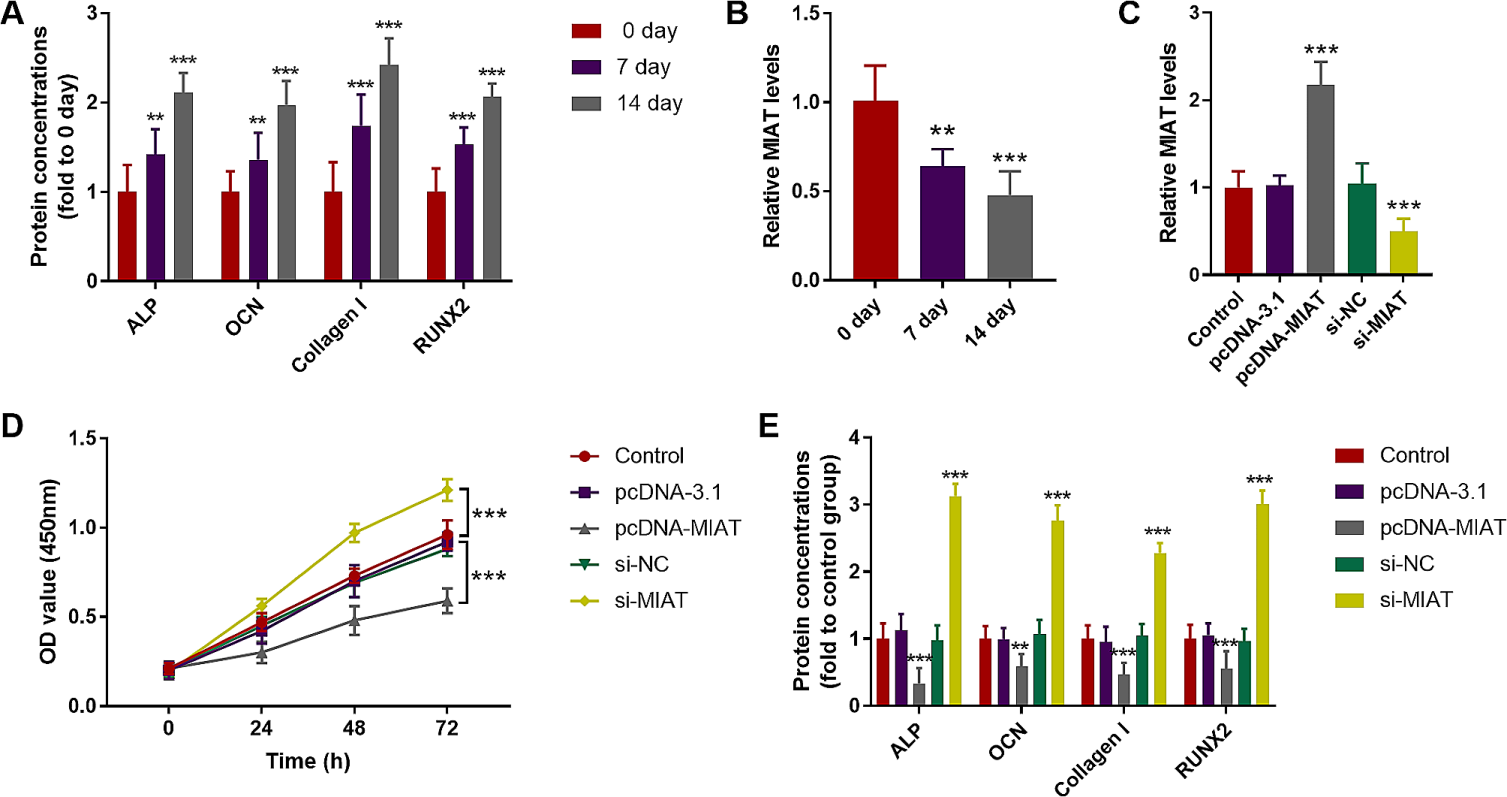
MIAT knockdown promotes osteogenic differentiation. **A**. Concentrations of ALP, OCN, Collagen I, and RUNX2 increased along with MC3T3-E1 cell differentiation. **B**. lncRNA MIAT decreased gradually along with osteogenic differentiation. **C**. pcDNA-MIAT transfected contributed to the upregulation of MIAT, while si-MIAT led to the levels’ downtrend. **D**. MIAT overexpression suppressed cell viability, whereas MIAT knockdown had the opposite effect. **E**. MIAT overexpression inhibited the release of ALP, OCN, Collagen I, and RUNX2, while MIAT promoted their release. ** *P* < 0.01, *** *P* < 0.001

MC3T3-E1 cells were incubated with BMP-2 to induce cell differentiation. As time passed, concentrations of ALP, OCN, Collagen I, and RUNX2 increased, illustrating the successful induction of osteogenic differentiation (Fig. 2A). Consistent with the value changes of osteogenic differentiation biomarkers, a gradually decreasing trend of lncRNA MIAT was also detected along with osteogenic differentiation (Fig. 2B). Therefore, the role of lncRNA MIAT in osteogenic differentiation was explored in MC3T3-E1 cells. Figure 2C presented the expression changes of MIAT in MC3T3-E1 cells after cell transfection, it was found that pcDNA-MIAT transfected contributed to the upregulation of MIAT, while si-MIAT led to the levels’ downtrend, and the influences were significant (*P* < 0.05). Based on the CCK-8 assay results, MIAT overexpression suppressed cell viability, whereas MIAT knockdown had the opposite effect (Fig. 2D; *P* < 0.05). In terms of osteogenic differentiation biomarkers, MIAT overexpression inhibited the release of ALP, OCN, Collagen I, and RUNX2, while MIAT inhibition promoted their release (Fig. 2E; *P* < 0.05).

### MIAT negatively regulated miR-181a-5p expression by directly targeting

**Fig. 3:**
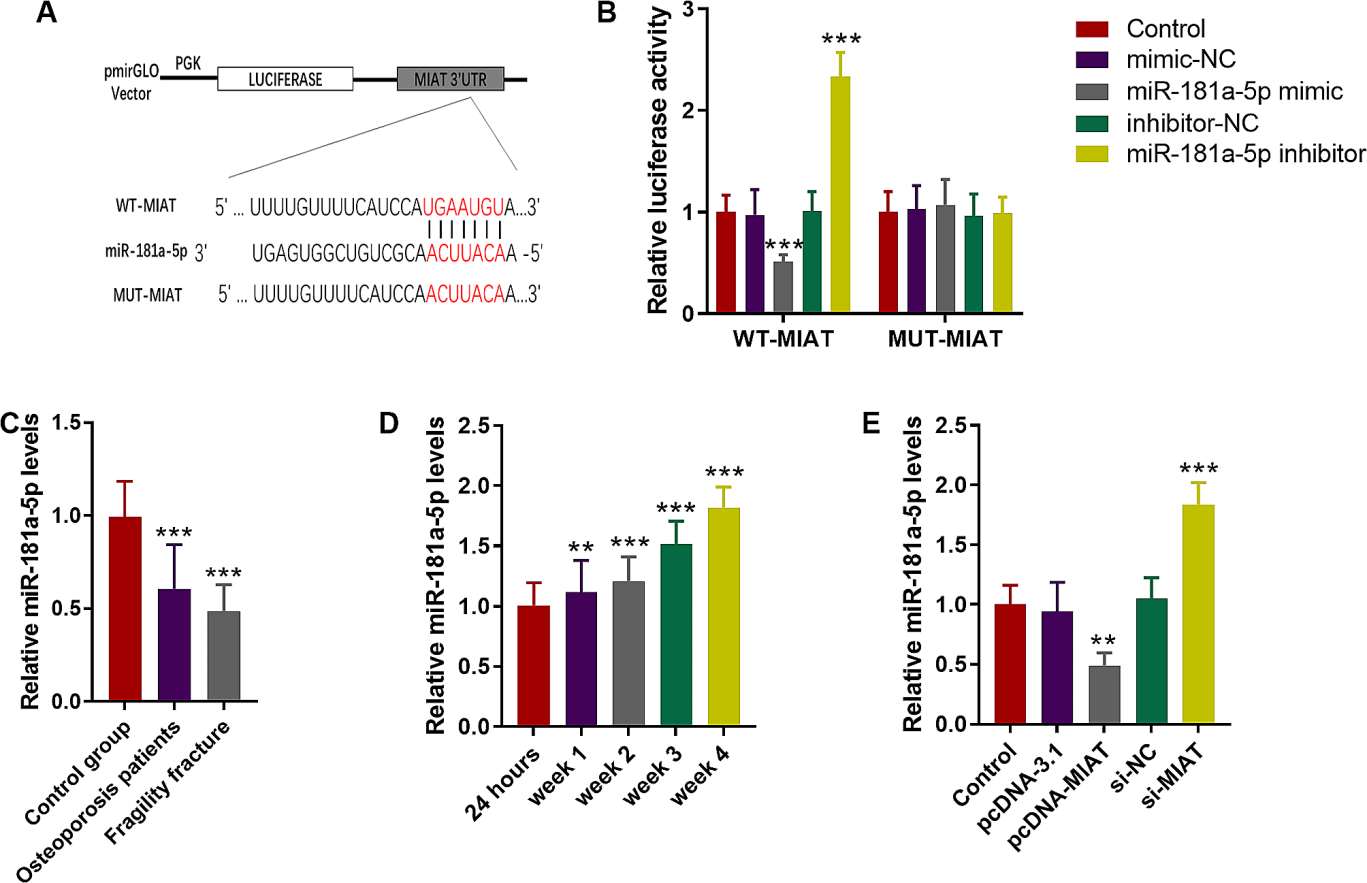
MIAT serves as a sponger of miR-181a-5p. **A**. The binding sites of miR-181a-5p to MIAT. **B**. Luciferase activity of cells after cell transfection in MC3T3-E1 cells. **C**. Serum miR-181a-5p was downregulated in osteoporosis and fragility fracture patients. **D**. After operation, serum miR-181a-5p increased gradually. **E**. In MC3T3-E1 cells, MIAT overexpression inhibited miR-181a-5p, while MIAT knockdown enhanced its expression. **P < 0.01; *** *P* < 0.001

Figure 3A presented the binding sites of miR-181a-5p to MIAT (Fig. 3A). Luciferase reporter assay confirmed that miR-181a-5p inhibitor typically increased the luciferase activity of cells transfected with MIAT-WT (*P* < 0.05), whereas the luciferase activity of cells with MIAT-MUT was not affected by miR-181a-5p levels (*P* > 0.05, Fig. 3B). Clinically, a gradual downtrend of miR-181a-5p was detected in the serum of osteoporosis and fragility fracture groups compared to the control group with a significant difference (Fig. 3C, *P* < 0.05). During fracture healing, serum miR-181a-5p was upregulated gradually and reached its highest at 4 weeks after operation (Fig. 3D). In MC3T3-E1 cells, MIAT overexpression inhibited the expression of miR-181a-5p. while si-MIAT led to the upregulation of miR-181a-5p, and the influences were significant (Fig. 3E, *P* < 0.05).

### Silencing of miR-181a-5p reversed the effects mediated by si-MIAT on MC3T3-E1 cells

**Fig. 4:**
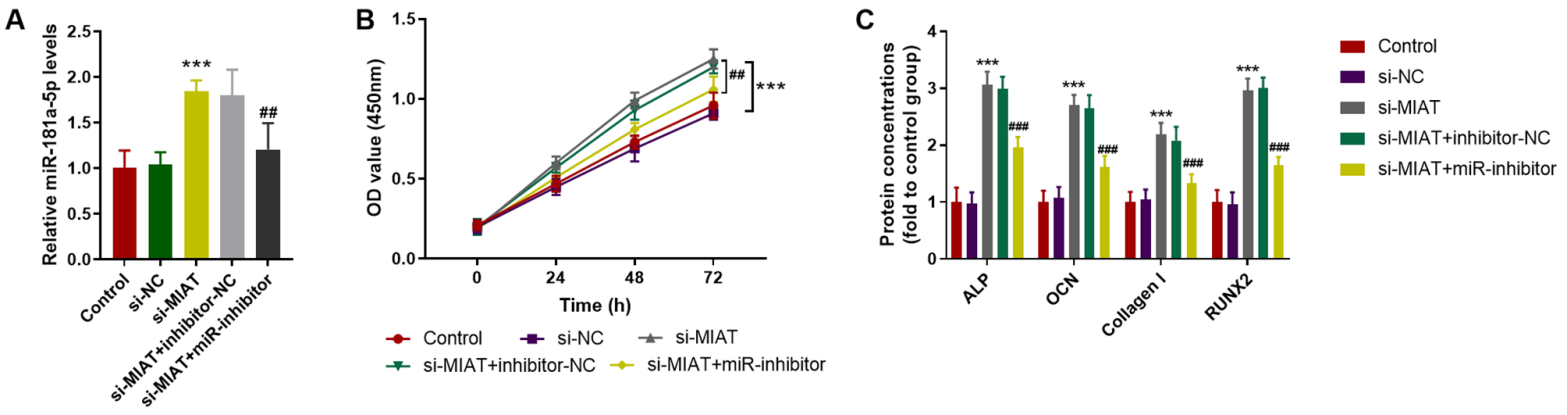
Silencing of miR-181a-5p reversed the effects mediated by si-MIAT on MC3T3-E1 cells. **A**. Increased miR-181a-5p levels caused by si-MIAT were remarkably attenuated by miR-181a-5p inhibitor. **B**. The restoration of cell viability induced by si-MIAT was partially mitigated by miR-181a-5p inhibitor. **C**. miR-181a-5p inhibitor also diminished the alleviation of osteogenic differentiation biomarkers by si-MIAT. *** *P* < 0.001 compared with control group; ## *P* < 0.01, ### *P* < 0.001 compared with si-MIAT group

To further verify the regulatory role of miR-181a-5p in MC3T3-E1 cells, miR-181a-5o inhibitor was transfected into cells. RT-qPCR results indicated that increased miR-181a-5p levels caused by si-MIAT were remarkably attenuated by miR-181a-5p inhibitor (*P* < 0.05, Fig. 4A). Besides, the restoration of cell viability induced by si-MIAT was partially mitigated by miR-181a-5p inhibitor (*P* < 0.05, Fig. 4B). Furthermore, miR-28-5p inhibitor also diminished the alleviation of osteogenic differentiation biomarkers by si-MIAT, presenting as the downtrend of ALP, OCN, Collagen I, and RUNX2 concentration (*P* < 0.05, Fig. 4C).

### Go and KEGG enrichment analysis

**Fig. 5:**
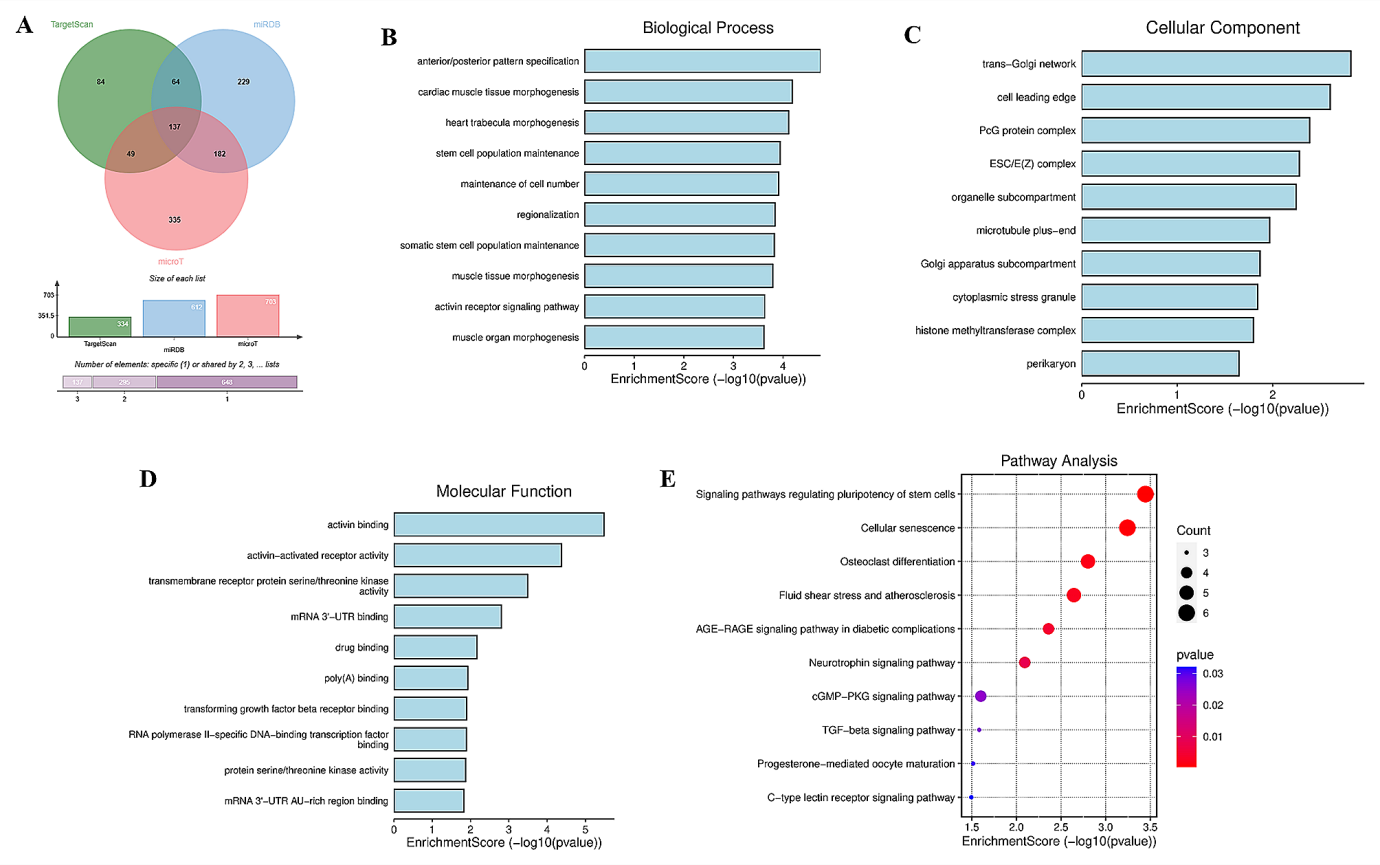
Go and KEGG enrichment analysis of miR-181a-5p target genes. **A**. Target genes of miR-181a-5p based on the miRDB, TargetScan and microT datasets. **B**. GO annotation results in terms of biological process. **C**. GO annotation results in terms of cellular components. **D**. GO annotation results in terms of molecular function. **E**. The top 10 enriched KEGG pathways

Figure 5A shows the predicted target genes of miR-181a-5p based on the miRDB, TargetScan and microT datasets. Results of target genes from the three databases were pooled to identify overlapping results. A total of 137 overlapping target genes were predicted by three databases, which were annotated via GO function and KEGG pathway analysis. The results of GO enrichment terms and KEGG pathway were summarized in Fig. 5B-E. As GO annotation results displayed, anterior/posterior pattern, specification cardiac muscle tissue, morphogenesis heart trabecula morphogenesis were included in the biological process (Fig. 5B), trans-Golgi network, cell leading edge, PcG protein complex were enriched in the cellular component (Fig. 5C), activin binding, activin-activated receptor activity, transmembrane receptor protein serine/threonine kinase activity were included in molecular function (Fig. 5D). Figure 5E presented the top 10 enriched KEGG pathways, in which signaling pathways regulating pluripotency of stem cells, cellular senescence, and osteoclast differentiation were mostly enriched.

## Discussion

LncRNAs are a class of non-protein-coding RNAs, and their differential expression in different cells or under different physiological and pathological conditions suggests their diagnostic function as a biomarker [15]. Based on the current findings, elevated levels of lncRNA MIAT were detected in the serum of osteoporosis patients. Moreover, ROC curve suggested the diagnostic value of serum MIAT for osteoporosis. Consistently, high values of MIAT have been determined in osteoporosis cases by Wang et al. [16]. Fragility fracture is the first symptom and reason for treatment in most patients with osteoporosis [17]. In the current study, patients with fragility fracture also had high values of MIAT compared with simple osteoporosis cases. In addition, we followed up on serum MIAT levels in patients with fragility fractures. It is known that lncRNAs can stably exist in serum samples, and are resistant to RNAase digestion. In addition, the preparation procedure of serum is simple, serum samples were used for research. Based on the results, it was found that patients’ MIAT levels gradually decreased during fracture healing. Therefore, it was inspired that the dynamic variable MIAT may be a key component during the occurrence and recovery of fragility fracture.

The abnormal expression of lncRNA is closely related to the occurrence and development of osteoporosis and related fractures [18]. A large amount of lncRNAs have been identified to be fundamental regulators of osteoblast differentiation, suggesting their useful role in the development to osteoblasts, such as ANCR and HOTAIR [19, 20]. Based on the present clinical data, lncRNA MIAT was detected to be highly expressed in the serum of osteoporosis patients, and a good diagnostic performance was shown for MIAT in differentiating osteoporosis. Consistently, elevated MIAT has been determined in the peripheral blood mononuclear cells of post-menopausal osteoporosis patients, which was related to the inflammatory response [12]. In addition, MIAT is also suggested to be a fundamental regulator of osteogenic differentiation [13]. Bone regeneration is critical for fracture healing, while osteogenic differentiation is considered to be a crucial aspect of bone regeneration [21]. In terms of the important role of MIAT in osteogenic differentiation, its role in fragility fracture and fracture healing was further investigated. As expected, serum MIAT levels were elevated in the fragility fracture group and decreased promptly during fracture healing. The findings illustrated the possible role of MIAT in fracture healing.

MC3T3-E1 cells are derived from mouse calvaria, serving as a well-studied pre-osteoblastic cell [22]. The biological function of MC3T3-E1 cells is the closest to that of primary cultured osteoblasts, which can be used to study the whole osteoblastic process of cells [23]. Under the current background of bone tissue engineering, MC3T3-E1 cells have been widely used to study the differentiation, proliferation and molecular mechanism of osteoblasts, and are the first choice of target cell types for many diseases. The main function of osteoblasts is to synthesize extracellular stroma and support mineralization [24]. Consistent with the activity of bone resorptive osteoclasts, this function is essential for bone remodeling and bone mass maintenance [25]. The imbalance of bone formation and bone resorption can lead to a variety of bone diseases, osteoporosis is one of them [26]. In the current study, MC3T3-E1 cells were applied for the exploration of the functional role of MIAT1 in osteogenic differentiation. It is known that siRNAs are useful in the gene function study, which can be used for therapeutic purposes [27–29]. In the current study, si-MIAT was determined to promote osteogenic differentiation, while overexpression MIAT1 had an adverse effect of the cell proliferation and differentiation. In hASCs, the promotive role of MIAT knockdown in cell differentiation was determined, which was in accordance with our present findings [13]. Thus, the results suggested that lncRNA MIAT may function as a suppressor in fracture healing. But only osteoblastic markers including ALP, OCN, Collagen I and RUNX2 was used to reflect the osteogenic differentiation in the current study, which have been determoined to be reliable markers for osteogenic differentiation [30, 31]. In future other different methods shold be used for the evaluation of osteogenic differentiation, such as Alizarin Red stain.

It is known that lncRNAs can regulate downstream target genes by regulating miRNAs at the transcription level. Moreover, the regulatory role of miRNA in several conditions has been studied, including the musculoskeletal system [32, 33]. In a study of osteoarthritis, MIAT is determined to be a competing endogenous RNA of miR-181a-5p [34]. Consistently, miR-181a-5p was also determined to be the target of lncRNA MIAT in MC3T3-E1 based on the present luciferase reporter assay. Previously, miR-181a-5p has been considered to be one of the mitochondria-associated microRNAs during the osteogenic differentiation of human MSCs [35]. A latest study also reports the high expression of miR-181a-5p during the osteogenic differentiation of MC3T3-E1 [35]. In fibrous dysplasia of bone, miR-181a-5p enhanced BMSC osteogenic differentiation [9]. Therefore, the involvement of miR-181a-5p in the role of lncRNA MIAT was explored in the present study. It was found that miR-181a-5p inhibitor reversed the promotive effect of MIAT knockdown on osteogenic differentiation.

In recent years, great attention has been paid to deciphering the regulatory mechanisms of miRNAs. By binding to target genes, miRNAs cause degradation of target gene mRNA or inhibition of their translation [36]. This is an important regulatory mechanism of miRNAs functionally. Thus, three online datasets were applied for the prediction of miR-181a-5p target genes. Finally, a total of 137 overlapping target genes were predicted by miRDB, TargetScan and microT datasets. Then the GO function and KEGG pathway analysis demonstrated these target genes were mainly enriched for terms related to signaling pathways regulating pluripotency of stem cells, cellular senescence, and osteoclast differentiation. The findings furthermore emphasized the important role of miR-181a-5p in bone metabolism. As reported, pharmacological strategies might influence the time and quality of bone healing [37]. Current methods of fracture care use various adjuncts to try and decrease time to fracture union, improve fracture union rates and enhance functional recovery, such as intermittent pneumatic compression (IPC) and physical stimulation [38, 39]. In light of the important role of MIAT/miR-181a-5p in osteogenic differentiation, its potential role in fracture healing deserves our attention. The present findings may provide possible new ideas for promoting fracture healing strategies.

## Conclusions

In conclusion, lncRNA MIAT serves as a promising biomarker for osteoporosis, and may participate in fracture healing. MIAT knockdown can promote osteogenic differentiation via targeting miR-181a-5p. This study can lead to the development of new diagnostic biomarker for osteoporosis and provides a new approach to study the pathogenesis of fragility fracture and fracture healing.

## Data Availability

The datasets used and/or analysed during the current study are available from the corresponding author on reasonable request.
